# The “Managed” or Damaged Heart? Emotional Labor, Gender, and Posttraumatic Stressors Predict Workplace Event-Related Acute Changes in Cortisol, Oxytocin, and Heart Rate Variability

**DOI:** 10.3389/fpsyg.2020.00604

**Published:** 2020-04-08

**Authors:** Arija Birze, Vicki LeBlanc, Cheryl Regehr, Elise Paradis, Gillian Einstein

**Affiliations:** ^1^Dalla Lana School of Public Health, University of Toronto, Toronto, ON, Canada; ^2^Department of Innovation in Medical Education, University of Ottawa, Ottawa, ON, Canada; ^3^Factor-Inwentash Faculty of Social Work, University of Toronto, Toronto, ON, Canada; ^4^Department of Sociology, University of Toronto, Toronto, ON, Canada; ^5^Department of Psychology, University of Toronto, Toronto, ON, Canada; ^6^Tema Genus, Linköping University, Linköping, Sweden

**Keywords:** police communicators, workplace stressors, emotional labor, gender role stress, posttraumatic stress, cortisol, oxytocin, heart rate variability

## Abstract

Vital to the everyday operation of police services, police communicators (911 call-takers and dispatchers) are persistently subject to imminent challenges in the workplace; they must always be prepared to engage and deal with a wide variety of circumstances that provoke various intense emotions and physiological stress responses. Acute changes in cortisol, oxytocin, and heart rate variability are central to adaptive responses in stressful complex social interactions, but they might also be indicative of physiological dysregulation due to long-term psychosocial stress exposures. Thus, we examine acute stress-induced release of peripheral oxytocin and cortisol along with changes in heart rate variability, and how each relates to persistent workplace stressors and symptoms of posttraumatic stress. Findings indicate chronic forms of gendered workplace stress such as emotional labor, gender role stress and, posttraumatic stress each have differential associations with, and predict physiological responses to, acutely stressful events in the workplace. These associations suggest potential mechanisms through which communicators become more vulnerable to developing stress-related disorders such as posttraumatic stress injuries, especially after cumulative traumatic exposures in this context. The results also suggest potential pathways for the biological embedding of stressful gendered workplace experiences.

## Introduction

Comprising^1^ mostly women, police communicators (911 emergency call-takers and dispatchers) are central to the everyday operations of emergency services. They are responsible for answering calls and dispatching services for both emergency and non-emergency calls from the public, as well as for internal service requests. As actual “first responders,” communicators are often exposed to similar incidents as those of frontline and special unit officers (Papazoglou, 2013; Andersen et al., 2016), repeatedly responding to events including violence, injury, death, and disaster, thereby increasing their risk of exposure to potentially traumatic events (Carleton et al., 2019). Police communicators are also subject to additional, environment specific stressors such as a close, confined workspace and controlled mobility, concentrated organizational scrutiny, intense focus and prolonged periods of rapidly paced work (Regehr et al., 2013), all of which may be understood as gendered aspects of their work (Acker, 1990, 1999, 2006).

The performance of their duties necessitates persistent social interactions with the public and officers in an effort to take control of the interactions and garner, triage, and transmit relevant information for positive outcomes (Regehr et al., 2013). Working within an organizational imperative to remain calm and emotionally neutral (Papazoglou, 2013), they are routinely required to negotiate a variety of emotions including anger, indifference, guilt, worry, sadness and helplessness. They must manage their own emotions while simultaneously managing the emotions of others (e.g., officers, callers, coworkers and supervisors) (Tracy and Tracy, 1998; Shuler and Sypher, 2000). When taking calls from the public, they work to gain control of the call, push their own feelings aside, calm the callers, retrieve accurate and relevant information, and quickly move on to the next call, often with extreme transitions in emotional valence (Whalen and Zimmerman, 1998). When dispatching calls to officers they work to organize and distribute resources effectively and efficiently and are responsible for the safety of the first responders they are managing, regardless of the potential dangers in every circumstance. Thus, emotional labor (EL), a well-supported sociological conceptualization of emotion-focused work requirements (Hochschild, 2003), is likely both an important aspect of the work of communicators and a source of chronic occupational stress.

A job is high in EL when it includes frequent interactions with the public, the management of ones’ own and others’ emotions, and where an organization has a vested interest in scrutinizing and enforcing the emotional landscape of the workplace (Wharton, 2009; Grandey et al., 2013; Grandey and Melloy, 2017). In order to present an appropriate display that falls in line with an organization’s feeling rules and societal norms, feelings either have to be suppressed, faked, changed or evoked by workers in an effort to elicit the desired response from others in the interaction. EL has been established as a multidimensional construct and techniques include cognitive, bodily and expressive changes that often co-occur (Hochschild, 1979; Erickson and Grove, 2008). Surface acting and deep acting are two EL strategies that have received considerable scientific attention (Wharton, 2009; Grandey and Gabriel, 2015). Both strategies are effortful processes that are meant to bring a worker’s emotional displays in line with organizational feeling rules, with the express purpose of managing the emotions of those with whom they are interacting. Surface acting leaves internal feelings intact as workers both hide unsuitable feelings and display feigned feelings that better fit the structural requirements of the job (Meanwell et al., 2008; Grandey and Melloy, 2017) – such as the masculinized expected absence of emotional expression found in policing cultures (Papazoglou, 2013). Deep acting involves working to change felt emotions so that they align with feeling rules and become more genuine in the performance (Meanwell et al., 2008; Grandey and Melloy, 2017). Although EL is consistently associated with emotional exhaustion, psychological strain and burnout (Hülsheger and Schewe, 2011), research has not generally examined the consequences for the many bodily systems that are regularly recruited to successfully accomplish work that has high demands for EL during uniquely stressful situations such as exposure to potentially traumatic events.

Exposure to traumatic events in the workplace varies significantly across occupations. Although communicators are not physically present at scenes, their distance may not buffer them from developing symptoms of posttraumatic stress (PTS) following exposure to traumatic incidents (Lilly and Pierce, 2013). The highest rates of exposure are regularly found in public safety personnel, including police communicators (Corneil et al., 1999; Regehr et al., 2004; LeBlanc et al., 2011; Regehr and LeBlanc, 2011; Berger et al., 2012; Pierce and Lilly, 2012; Skogstad et al., 2013; Klimley et al., 2018; Carleton et al., 2019). This higher rate of exposure often comes with sizeable implications for the health and well-being of workers. Increased rates of depression, anxiety, and PTS are the norm (Berger et al., 2012; Lilly and Pierce, 2013; Klimley et al., 2018). Furthermore, symptoms of PTS are regularly associated with changes in physiological stress response (Regehr et al., 2007; Pacella et al., 2013; van Zuiden et al., 2013; Mouthaan et al., 2014; Morris et al., 2016; Pyne et al., 2016; Regehr and LeBlanc, 2017) and with physiological dysregulation over the long-term (McEwen, 2002; Glover et al., 2006). Despite having been identified as having a particularly elevated prevalence of PTS (Regehr et al., 2013; Regehr and LeBlanc, 2017), little research has considered the acute stress response and its associations with work-related stressors in this often gendered and civilianized population (Meischke et al., 2015; Klimley et al., 2018; Steinkopf et al., 2018).

Biological embedding of stressful, gendered life experiences is one example of how the social becomes biological (Freund, 2006; Einstein, 2012). For instance, although men report higher rates of exposure to traumatic events, women develop posttraumatic stress disorder (PTSD) at significantly higher rates (Dückers and Olff, 2017). Evidence suggests that these gender disparities in the development of PTSD can be explained by qualitative differences in the nature of traumatic exposures (Silove et al., 2017), including within occupations such as policing (Gehrke and Violanti, 2006). Exposure to potentially traumatic events in the workplace is, thus, one domain where these differences play out. It is perhaps not accidental that highly emotion-focused professions are often feminized, predominantly employing women, thereby leading to differentiated traumatic exposures and workplace expectations that are characterized by elevated emotional demands (Acker, 2006; Wharton, 2014). Thus, varying social role expectations regarding appropriate responses to traumatic exposure, along with expectations for caring for others during a shared trauma, may lead to uniquely gendered stress for communicators. Specifically, while communications work is often organized as primarily women’s work and is recognized as requiring EL (Tracy and Tracy, 1998), communicators are still expected to espouse a masculinized emotional approach to their intensely emotional work.

As a function of gendered socialization, individuals learn to engage in social interactions in particular ways that are linked to their biological sex and generally understood as either appropriately feminine or masculine ways of being (Ridgeway, 2009; Risman, 2018). Through these socialization processes women and men are held accountable, both by themselves and others, to conform to these norms. Among those who feel both pressures to conform to appropriate norms and have a rigid commitment to these socially prescribed ways of being, situations that threaten their gendered sense of self can be particularly distressing. Thus, feminine and masculine gender stress (GS) are measures of perceived distress that are experienced in situations that are discordant with one’s closely held gender beliefs, including in the workplace (Eisler and Skidmore, 1987; Gillespie and Eisler, 1992). For instance, for men working in feminized occupations, there is a stronger relationship between masculine ideology and social stress or psychological strain compared to men in masculinized occupations (Sobiraj et al., 2015). For women working in masculinized occupations – such as policing – having a masculine gender identity is associated with lower levels of job satisfaction compared to women who have either feminine or androgynous gender identities (Swan, 2016). As such, conforming to widely held stereotypical gender norms, including appropriate emotional presentations, can be a persistent day-to-day stressor that is also associated with other work-related stressors like EL. Earlier research linking GS in occupations to physiological stress found that in a male dominated service delivery organization, although men had higher masculine GS than women, higher masculine GS was associated with higher blood pressure in both men and women employees (Watkins et al., 1991). What remains to be seen is whether GS is associated with other chronic work-related stressors such as EL requirements and distress related to traumatic exposures, as well as acute physiological stress responses in the workplace.

The release of cortisol during acute stress – or what has historically been referred to as the fight or flight response – has been shown to predict both general stress levels during everyday life (Kidd et al., 2014), distress and resilience (Galatzer-Levy et al., 2014), future health outcomes (Hamer and Steptoe, 2012), and the potential for progressively developing PTSD (McFarlane et al., 2011; van Zuiden et al., 2013). More recently, some have proposed a theoretical broadening of acute stress processes to include additional affiliative or calm and connect responses, especially in women, during times of threat, called: “tend and befriend” (Taylor, 2006). The neuropeptide oxytocin is thought to be central to this ostensibly more “prosocial” biobehavioral response (Taylor, 2006; von Dawans et al., 2019). Oxytocin release during stressful situations has not thus far been examined in relation to health outcomes but researchers are beginning to explore its association with stress regulation (Olff et al., 2013), emotional trauma (Donadon et al., 2018) and PTSD (Frijling et al., 2015). Exploring the role of oxytocin response in the context of potentially traumatic exposures in the workplace has the potential to further expand our understanding of these broader conceptions of acute stress response.

The oxytocin system is thought to have a reciprocal relationship with the hypothalamic-pituitary-adrenal (HPA) axis which is responsible for the release of cortisol (Feldman, 2012). The release of oxytocin is thought to buffer or dampen HPA activity during acute stress (Neumann, 2008; Olff et al., 2013), improve accuracy for emotion perception (Fischer-Shofty et al., 2013), and support emotion regulation (Quirin et al., 2011). Some recent evidence suggests that the overarching role of oxytocin is to aid in social adaptation by increasing the salience of social cues, allowing it to either facilitate or attenuate stress depending on the social context (Shamay-Tsoory and Abu-Akel, 2015). Only very recently have researchers begun to demonstrate that, in the short term, cortisol and oxytocin release are positively related following psychosocial challenges (Engert et al., 2016; Alley et al., 2019). These findings suggest that oxytocin is important for work-related traumatic exposures, especially with respect to emotion work requirements, but the specificity of oxytocin’s role in the experience of complex social stress and traumatic exposure is not well understood. Furthermore, recent evidence suggests some efficacy for oxytocin in the prevention and intervention of posttraumatic stress symptoms (Frijling, 2017; van Zuiden et al., 2017; Koch et al., 2019).

Neuroendocrine responses to stress will then activate changes in the autonomic nervous system (ANS), including both the parasympathetic nervous system (PNS) and the sympathetic nervous system (SNS). Heart rate (HR) is determined by the integration of these various regulatory systems required for successful adaptation to the environment, including psychosocial challenges (Thayer and Lane, 2009; Shaffer and Ginsberg, 2017). Heart rate variability (HRV), the flexible time intervals between successive heartbeats, is thought to be an index of neurocardiac function that reflects the dynamics of the ANS and interactions between the brain and heart (Shaffer et al., 2014). Generally speaking, at rest the PNS or vagal activity dominates, slowing HR and increasing HRV. Under stress, the SNS dominates, increasing HR and decreasing HRV. While increases in HRV due to vagal stimulation are almost immediate, reductions in HRV due to sympathetic activation are delayed by up to 5 s (Shaffer et al., 2014).

Heart rate variability has also recently been conceptualized as a psychophysiological marker of health and wellbeing that acts as a momentary indicator of capacity for flexibly responding to stressful social engagements (Thayer and Lane, 2009; Kemp et al., 2017). More specifically, and similarly to oxytocin, HRV has been associated with emotional flexibility with respect to regulating one’s own emotional state and response to others’ (Appelhans and Luecken, 2006; Tuck et al., 2016), as well as the ability to perceive the emotional state of others (Quintana et al., 2012). In women police officers, lower resting HRV is associated with greater perceptions of lack of organizational support (Andrew et al., 2017), suggesting links with more chronic, ongoing relational and organizational stressors as well. Contrary to conventional understanding, however, a recent review found that several studies investigating phasic changes in HRV or cardiac vagal control have shown *increases* in HRV when individuals are subject to variably stressful conditions requiring emotion regulation (Balzarotti et al., 2017).

Thus, each of these interconnected systems – the HPA axis, the oxytocinergic and the ANS – are central to social stress processes and contribute to adaptive functioning in times of complex social stress (Norman et al., 2012), making them relevant for investigation in workplace contexts involving persistent emotion work requirements and exposure to potentially traumatic events. Only recently have researchers begun to examine some of the associated physiological responses (cortisol) to performing EL (Lim et al., 2018). However, given the relational basis of communicators’ workplace stressors (Shuler and Sypher, 2000; Mann, 2004), and oxytocin’s importance for social interaction (Shamay-Tsoory and Abu-Akel, 2015), exploring oxytocin’s role in acute psychosocial stress response has the potential to shed new light on the relationship between systemic dysregulation or poor health and emotion work requirements. Whether it facilitates or attenuates stress during persistent social engagement and demanding emotion work during acutely stressful events has yet to be explicated. Additionally, little work has been done on (a) the integrated response of cortisol, oxytocin and HRV during times of stress and (b) how these acute physiological responses might relate to subjective experiences of chronic workplace stressors.

There is accumulating evidence that chronic psychosocial stressors contribute to the development of posttraumatic stress injuries (Brooks Holliday et al., 2018). However, research examining the various patterned sources of psychosocial stressors and their underlying pathways is in its infancy. Although research using short-term laboratory stressors has been foundational to understanding stress physiology, the lack of fidelity to life’s circumstances has undermined the relative importance of complex social structures, interactions and their lasting repercussions. The study of chronic and patterned psychosocial stressors and how they shape acute stress response needs further examination (Chida and Hamer, 2008), especially in naturalistic settings.

Accordingly, we undertook an exploratory study of chronic and acute workplace stress in police communicators in a naturalistic setting, their communications workplace. We hypothesized that in this setting, cortisol, oxytocin, and HRV response to acute workplace stressors would be associated with one another and that they would each be differentially associated with chronic subjective work-related stressors, suggesting their importance for understanding both short and long-term consequences of communicators’ work. To test this hypothesis, we assessed oxytocinergic, HPA axis, and ANS activity through repeated sampling of salivary oxytocin and cortisol, and by recording HRV during stressful, yet common events. We then related the physiological changes following acutely stressful workplace events, to chronic psychosocial stressors including EL, GS, and PTS. Our intention was to evaluate the respective contributions of the various chronic workplace stressors to the variance of physiological response during acute events.

## Materials and Methods

This study is part of a larger mixed methods project on gendered experiences of occupational stress and how they might shape the biology and long-term health outcomes of police communicators. Ethics approval was obtained from the University’s Research Ethics Board and permission to conduct this research onsite was granted by the police service. The research was carried out, on site, in a large urban police communications center.

Multiple visits to the service – comprising 400 h over the course of 9 months – were made for a total recruitment of 81 participants (65 self-identified women and 16 self-identified men). Upon consent, arrangements were made for individual participation, where the researcher scheduled one observation session with each participant, during the course of a regular working day. Each individual data collection and observation session lasted approximately 3 to 4 h. At the beginning of these sessions, each participant was outfitted with a HRV monitor and the researcher was prepared to collect additional physiological data (salivary oxytocin and cortisol) should an acutely stressful event occur in real-time, during the session. At the end of each session, the participants were given a set of questionnaires to be completed at home, described in section “Subjective Self-Report Stressors” below.

Over the course of the 81 individualized sessions, a number of potentially stressful events took place as communicators conducted their everyday duties. Participants’ verbal and behavioral responses determined which events might be stressful, as they occurred. For example, statements like “oh, he’s got a knife, here we go,” “OK, I need to calm down,” “that’s our ultimate stress,” and explicatives, on the part of the responders, were viewed as marking acutely stressful moments. Physical responses like trembling hands or sudden changes in posture and attention, were also viewed as marking a stressful situation. Across the 81 sessions, 25 participants were identified as having potentially acutely stressful events while they were either call-taking or dispatching. Details of the events and their timing were then noted by the researcher and saliva samples were collected for oxytocin and cortisol analyses at 20, 30, and 40 min post-event onset. Acutely stressful events occurred during both day and night shifts. The 25 events included: possible overdose deaths, imminent suicides, people with weapons, witnesses to extreme violent crimes, shootings, coordination of and dispatching for complex fire scenes including burn victims and deaths, officers on scene where weapons were present, losing contact with officers, and dispatching officers to potentially dangerous unknown trouble calls or crimes in progress such as break-and-enters.

### Subjective Self-Report Stressors

#### Emotional Labor Scale – Revised (ELS-R)

The ELS-R is job-focused and measures the perceived duration, variety, frequency, and intensity of emotional expression, faking and hiding on an average workday (Brotheridge and Grandey, 2002; Brotheridge and Lee, 2003). Convergent and discriminant validity are supported (Lee and Brotheridge, 2011) and Cronbach’s alphas for the subscales range from 0.69 to 0.95 (Lee et al., 2010).

#### Gender Role Stress Scale (GRSS)

We administered both the Masculine Gender Role Stress Scale (MGRSS) and the Feminine Gender Role Stress Scale (FGRSS) to all participants. To reduce gendered social desirability biases we combined the masculine and feminine stress scales to form a long-form gender role stress scale. Items were not explicitly distinguished as either feminine or masculine, but rather, allowed participants to respond without being primed about behaviors stereotypically labeled as belonging to one gender group. The MGRSS assesses the stress that results from responding to situations that are in direct conflict with traditional ideals of masculinity, including, for example “having others say that you are too emotional” (Eisler and Skidmore, 1987). Reliability and validity are reported as adequate and Cronbach’s alpha for total score is 0.91 (Bekker and Boselie, 2002). The FGRSS scale assesses the stress that results from responding to situations that are in direct conflict with traditional ideals of femininity, including, for example, “having others believe that you are emotionally cold” (Gillespie and Eisler, 1992). Cronbach’s alphas for the subscales range from 0.77 to 0.83 and the total score has an alpha of 0.93 (Richmond et al., 2015).

#### Impact of Event Scale-Revised (IES-R)

The IES-R measures subjective distress following traumatic events (e.g., “I found myself acting or feeling like I was back at that time”; Weiss et al., 1995). Good concurrent and predictive validity are reported (Creamer et al., 2003). This scale also has high internal consistency with Chronbach’s alphas for subscales and total score ranging from 0.81 to 0.95 (Regehr et al., 2013).

### Acute Cortisol, Oxytocin, and HRV Sampling

During observations, saliva samples were taken from participants who were experiencing a potentially stressful event (*n* = 25). Using Sarstedt salivettes (Sarstedt, Germany), samples were taken at 20, 30, and 40 min after the event began, regardless of whether the event was ongoing or resolved. There can be wide intra- and interindividual variations in response to psychosocial challenge (Zänkert et al., 2018), but peak salivary oxytocin and cortisol generally occur between 10 and 40 min post-event onset (de Jong et al., 2015; Schladt et al., 2017). Healthy recovery from acute stressors generally results in a return to baseline levels 40 to 60 min after the stressor terminates (Kemeny, 2003). After collection, samples were immediately placed on ice, transferred to a freezer and stored at −20°C until they were shipped on dry ice for enzyme immunoassay (lab of Dr. Sue Carter, Kinsey Institute, Indiana University). In this field setting it was important to remain as minimally invasive as possible. Therefore, participants were allowed to eat and drink according to their preferred mealtimes, before their participation session had begun. However, once their participation session started, all were asked to abstain from eating and drinking anything, other than the occasional sip of water. If an event occurred, participants did not have anything by mouth until their saliva samples were completed.

Participants also wore a Firstbeat BodyGuard2 (Parak and Korhonen, 2013) HRV recording device during their observation period. Recordings were analyzed using the Kubios HRV Standard (ver. 3.1.0.1) software package. The first 30 s from each event were extracted from the full-length recording. While there are many HRV variables, we chose to use the square root of the mean squared differences between successive RR intervals (RMSSD), which reflects the beat-to-beat changes mediated by the PNS. It is highly correlated with high frequency-HRV (HF) but is less affected by changes in breathing frequency compared to HF-HRV and is acceptable for short-term recordings (Kemp et al., 2017), making it suitable for ambulatory measurements.

### Data Analyses

To assess overall output levels, as well as reactivity over time, area under the curve with respect to ground (AUC_G_) and with respect to increase (AUC_I_) calculations were completed for both cortisol and oxytocin levels using the three samples collected at 20, 30, and 40 min post-event onset, with formulas outlined by Pruessner et al. (2003). Both AUC_G_ and AUC_I_ are of interest as distinct assessments (Pruessner et al., 2003; Khoury et al., 2015). AUC_G_ is understood as a measure of overall intensity, capturing regularly circulating hormone levels as well as anticipatory stress and a singular acute response. AUC_I_ is understood to measure only reactive increases and/or decreases over a finite period of time, thereby eliminating any measurement of regularly circulating hormone levels as well as anticipatory stress (Engert et al., 2013; Khoury et al., 2015). Correlational analyses were used to test for associations between subjective self-reports of chronic workplace stressors and both AUC_G_ and AUC_I_ variables for oxytocin and cortisol. AUC_I_ values for both oxytocin and cortisol were unrelated to any of the demographic characteristics or self-report assessments. As such, AUC_I_ values were dropped from further analyses.

To address the relative independence and importance of chronic workplace stressors for physiological response, variables that either approached or had significant correlations (any *p* < 0.10) were included in a series of multiple regressions, with EL, PTS, and GS simultaneously entered into the regression equations to predict acute physiological responses. Three multiple linear regressions were calculated to predict oxytocin (AUC_G_), cortisol (AUC_G_), and RMSSD during an acutely stressful event. Cases were excluded pairwise to address missing values.

## Results

Twenty-two women and three men experienced potentially stressful events during their participation sessions. Of those who experienced an event, the average age was 36.5 years and the average time on as a communicator was 8 years. Four women reported using birth control and one reported hormone replacement therapy. Sample demographics and descriptive statistics for both subjective assessments of workplace-related stress and physiological responses to acute events are reported in Table 1. Although the range of the physiological variables is large, because of the limited sample size, we were unable to control for these potentially confounding variables. Additionally, it should be noted that, while the mean score on the IES-R is 25.57, using the more conservative cut-off of 33, suggested by Creamer et al. (2003), resulted in 35% of this sample reporting symptoms of PTS that met the criteria for PTSD.

**TABLE 1:** Sample demographics and descriptives.

	**Minimum**	**Maximum**	**Mean**	**SD**
Age	25	49	36.54	7.84
Years as a comm.	0.33	25	7.89	7.09
IES-R	0	59	25.57	18.65
FGRSS	39	148	92.72	28.07
MGRSS	21	103	59.36	21.57
Deep Acting (ELS-R)	3	13	8.36	2.46
Surface Acting (ELS-R)	11	22	17.38	3.28
Cortisol AUC_G_ (nmol/L)	45.42	155.83	103.96	32.68
Cortisol AUC_I_ (nmol/L)	−48.49	49.60	5.83	19.58
Oxytocin AUC_G_ (pg/mL)	92.10	190.39	127.84	31.17
Oxytocin AUC_I_ (pg/mL)	−35.93	37.51	1.32	14.19
RMSSD (ms)	5.9	56.3	17.83	11.67

*IES-R, Impact of Event Scale – Revised; FGRSS, Feminine Gender Role Stress Scale; MGRSS, Masculine Gender Role Stress Scale; ELS-R, Emotional Labor Scale – Revised; AUC_G_, area under the curve with respect to ground; RMSSD, root mean square of successive heartbeat interval differences.*

### Acute Cortisol, Oxytocin, and HRV and Associations With Chronic Subjective Stressors

**FIGURE 1:**
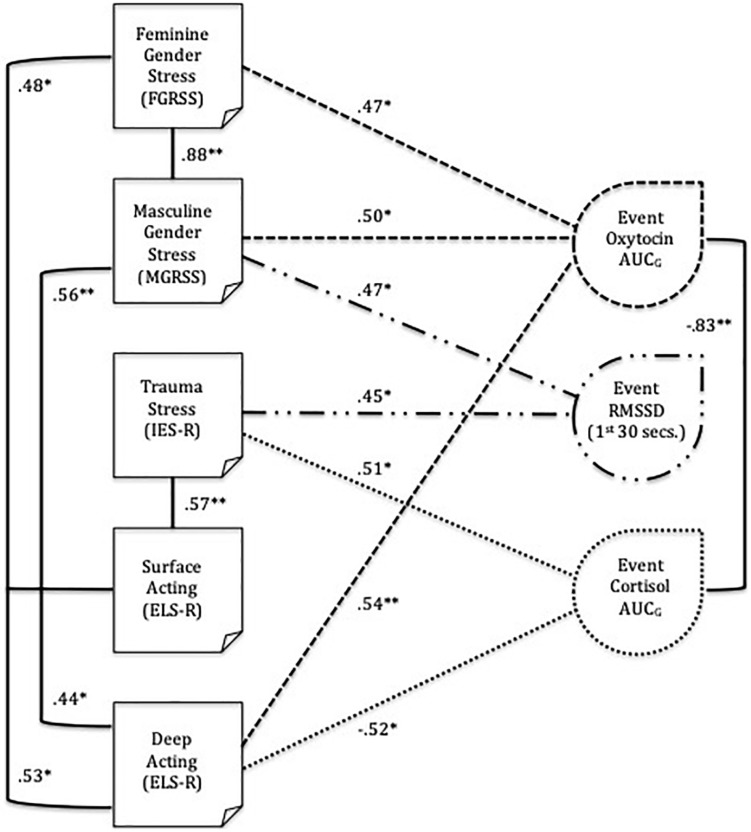
Bivariate correlations of chronic stressors (gender stress, posttraumatic stress, and emotional labor) with acute oxytocin and cortisol output and HRV after a stressful workplace event. ** is significant at the 0.01 level, * is significant at the 0.05 level. ELS-R, Emotional Labor Scale-Revised; IES-R, Impact of Event Scale-Revised; RMSSD, root mean square of successive differences; AUCG, area under the curve with respect to ground.

There were a number of significant correlations among subjective stressors and physiological responses (Table 2). Coefficients are displayed using a visual representation of the correlations in Figure 1. Cortisol (nmol/L) and oxytocin (pg/mL) AUC_G_ levels had a strong negative correlation with each other, but both were unrelated to RMSSD (ms). Cortisol, oxytocin, and RMSSD had significant associations with a number of ongoing stressors, namely GS, EL, and PTS. Oxytocin had a significant positive correlation with both feminine and masculine GS and deep acting, whereas cortisol had a significant positive association with PTS and a significant negative association with deep acting. RMSSD had a significant positive association with masculine GS and PTS.

**TABLE 2:** Correlation coefficients for subjective stressors and physiological stress responses.

	1.	2.	3.	4.	5.	6.	7.	8.
(1) FGRSS	–							
(2) MGRSS	0.88**	–						
(3) IES-R	0.07	0.12	–					
(4) Surface Acting (ELS-R)	0.48*	0.56**	0.57**	–				
(5) Deep Acting (ELS-R)	0.53*	0.44*	0.06	0.38	–			
(6) Oxytocin AUC_G_ (pg/mL)	0.47*	0.50*	−0.38	0.13	0.54**	–		
(7) Cortisol AUC_G_ (nmol/L)	−0.22	−0.25	0.51*	0.11	−0.52*	−0.83**	–	
(8). RMSSD (ms)	0.34	0.47*	0.45*	0.32	−0.38	−0.17	0.35	–

*FGRSS, Feminine Gender Role Stress Scale; MGRSS, Masculine Gender Role Stress Scale; IES-R, Impact of Event Scale – Revised; ELS-R, Emotional Labor Scale – Revised; AUC_G_, area under the curve with respect to ground; RMSSD, root mean square of successive heartbeat interval differences.*

### Chronic Subjective Stressors Predict Acute Cortisol, Oxytocin, and RMSSD

Three multiple linear regressions were run with subjective experiences of work-related stressors as predictors if they were found to have significant or, approaching significant, correlations with cortisol, oxytocin, or RMSSD.

The first multiple linear regression model predicted a significant amount of variance in cortisol output [*F*(2,19) = 9.771, *p* < 0.001) with an *R*^2^ of 0.507 (Table 3). Deep acting and PTS were both unique and significant predictors of overall cortisol output during the stressful events. A higher rate of PTS and lower reported deep acting predicted higher cortisol levels.

**TABLE 3:** Standardized Beta coefficients for predicting Cortisol AUCG levels during an acutely stressful event.

	**ß**	***t***	***p***
Deep acting (ELS-R)	− 0.493	− 3.060	0.006
IES-R	0.489	3.030	0.007

**Standardized Beta coefficients for predicting Oxytocin AUCG levels during an acutely stressful event**			

	**ß**	***t***	***p***
Deep acting (ELS-R)	0.399	2.557	0.020
IES-R	− 0.419	− 2.764	0.013
GRSS	0.444	2.822	0.011

**Standardized Beta coefficients for predicting first 30-second RMSSD during an acutely stressful event**			

	**ß**	***t***	***p***
IES-R	0.408	2.130	0.048
MGRSS	0.423	2.208	0.041

*ELS-R, Emotional Labor Scale – Revised; IES-R, Impact of Event Scale – Revised; GRSS, Gender Role Stress Scale; MGRSS, Masculine Gender Role Stress Scale.*

The second multiple linear regression model predicted a significant amount of variance in oxytocin output [*F*(3,18) = 8.866, *p* < 0.001) with an *R*^2^ of 0.596 (Table 3). GS, deep acting and PTS were all unique and significant predictors of overall oxytocin output. Higher levels of GS, more deep acting and fewer PTS symptoms predicted higher oxytocin levels.

The third multiple linear regression model predicted a significant amount of variance in event-related RMSSD [*F*(2,17) = 5.266, *p* < 0.017) with an *R*^2^ of 0.383 (Table 3). Both PTS and masculine GS were unique and significant predictors of RMSSD. Higher PTS and masculine GS predicted higher RMSSD during a stressful event.

## Discussion

The purpose of this exploratory study was to better understand chronic and gendered psychosocial stressors and how they might shape acute stress response in a population of police communicators, while they were working. In this particular workplace and for this type of work, we found that self-reported EL, GS, and PTS each independently predicted acute oxytocin and cortisol output as well as HRV, in different ways. Specifically, less deep acting and more PTS predicted greater cortisol output, more deep acting and GS, as well as less PTS, predicted greater oxytocin output, and finally, more PTS and masculine GS predicted higher HRV in the first 30 s of an event. Taken together, these findings speak to how sources of chronic workplace stressors (EL, PTS, GS) may relate to acute physiological stress responses (cortisol, oxytocin, and HRV) in ways that potentially worsen workers’ health over the long-term. Of particular importance is that these gendered stressors played a role in the acute activation of the physiological responses. We believe that this is the first study to report on the relationship between gendered psychosocial stressors and physiological stress responses in a workplace setting. Exploring these kinds of relationships is important for gaining a better understanding of the components of gendered work and workplaces that need to be considered to ensure the long-term health of workers.

### Emotional Labor

Our finding that deep acting is positively related to oxytocin AUC_G_ could be interpreted as showing both the protective effects of deep acting (Xanthopoulou et al., 2018) and the cardioprotective effects of oxytocin, against the harmful effects of social stress, especially among women (Koenig and Thayer, 2016). Although EL is consistently associated with feminized work, ill-health, emotional exhaustion, anxiety and depression (Brotheridge and Grandey, 2002; Guy and Newman, 2004; Mann, 2004), it is not always found to have negative impacts on workplace wellbeing. Some research has demonstrated that deep acting and the resulting genuine emotional displays can sometimes be profoundly rewarding and protective (Hülsheger et al., 2015; Xanthopoulou et al., 2018). Additionally, cognitive reappraisal processes, an adaptive coping strategy of lessening the emotional impact of a stressful situation by reframing or reappraising the initial perception of it, might closely align with deep acting (Grandey and Melloy, 2017). Both have been associated with more adaptive stress responses; cognitive reappraisal with reduced cortisol reactivity (Campbell and Ehlert, 2012), and deep acting with greater sense of personal accomplishment (Hülsheger and Schewe, 2011). Furthermore, Koenig and Thayer (2016) suggest that oxytocin plays a role in women’s higher vagal control of the heart, and that these systems would appear to support the tend-and-befriend model of stress response. This model would presumably include trying to bring personal feelings in-line with those of one is befriending (deep acting) in an effort to improve social bonds.

Nevertheless, additional evidence calls the protective effects of deep acting into question. Deep acting has also been associated with physical and psychosomatic complaints (Hülsheger and Schewe, 2011). It may be that there are hidden costs for the body from deep acting; somatization-related complaints rather than psychological strain. Interestingly, recent theorizing around oxytocin response suggests the oxytocin stress-regulation system is a likely biological mechanism for somatization-related complaints (Seng, 2010). Furthermore, as Hochschild (2003) has suggested, EL can have negative effects on health and wellbeing, regardless of the chosen strategy. Evidence suggesting any hidden physiological costs of deep acting is appropriately cautionary against assuming that deep acting and oxytocin are inherently protective. The positive correlation between oxytocin and deep acting under conditions of high social stress may underpin and compound potentially negative psychosomatic effects through the accumulation of gendered experiences throughout life, such a care- or service-based work that requires persistent EL.

The negative effects of EL may also be compounded when the organization fails to recognize EL as integral to the work of daily navigation of emotional extremes. In a qualitative study of police communicators, Shuler and Sypher (2000) found the organizational imperative of remaining emotionally neutral worked to pathologize rather than validate and support the very real emotional experiences of workers. Our finding of a positive association between surface acting and PTS would appear to support this argument and provide evidence that suggests an important link between EL strategies and PTS in this line of work. A potential explanation is that when experiencing emotional symptoms from traumatic experiences, emotional states fall farther from organizational feeling rules, thereby making deep acting more difficult to accomplish. Indeed, evidence suggests a link between emotional labor strategies and posttraumatic stress and burnout among public safety workers (Schaible and Gecas, 2010; Park et al., 2018). Furthermore, our finding of a negative association between deep acting and cortisol AUC_G_ also supports this argument, by demonstrating that communicators who report less deep acting have greater cortisol responses during stressful events. Surface acting may then become the more possible or necessary strategy. In this case, it is possible that those suffering from trauma symptoms find it necessary to mask their emotional responses in an effort to dampen emotional arousal (Jakupcak et al., 2006). Perhaps not surprisingly, those with impaired emotion regulation or an inability to down-regulate negative affect, experience greater increases in cortisol to a public speech task unless they’ve been administered intranasal oxytocin (Quirin et al., 2011). For the communicators in the present study, greater oxytocin response during acutely stressful workplace events appears to facilitate the completion of job requirements through sustained efficiencies in deep acting or emotion recognition and regulation. But at what cost? This association, in conjunction with the positive relationship between oxytocin and both feminine and masculine GS, as discussed in the following section, appears to suggest further potential for hidden costs to the body. The organizational imperative to remain emotionally neutral may have unintended consequences for both communicators and those with whom they interact.

### Gender Stress

We found that both feminine and masculine GS were positively related to oxytocin AUC_G_ and both EL strategies of surface and deep acting. As such, oxytocin may be functioning as a physiological indicator of social distress surrounding efforts to conform to closely held normative gender ideals, perhaps through increased EL strategies. Although higher oxytocin has repeatedly been associated with reduced cortisol responsivity (Neumann and Slattery, 2016), there also exists a substantial amount of research suggesting that oxytocin operates as a physiological marker of social and relationship distress (Taylor, 2006). The gender stress scales used in this study assess levels of distress experienced as a result of having your valued gender norms threatened. Because emotional interactions are closely tied to gender norms, many of the questions pertain to the emotional relationships respondents have with others or with the likeliness of emotional distress in response to certain social interactions that might be distressing through the *salience* of gender (e.g., “talking with someone who is angry with you” or “being perceived as having feminine traits” or “having others say that you are too emotional”; Eisler and Skidmore, 1987; Gillespie and Eisler, 1992). This suggests that gender role stress is also an indicator of higher levels of relational distress or social distress more generally. In our study, then, basal levels of gendered relational distress are predictive of oxytocin response. This is peripherally supported by additional research linking oxytocin with strained affiliations. Higher oxytocin is associated with relational distress in women who articulate a response to a friend/partner/relative who has recently committed a relational transgression (e.g., romantic infidelity, social rejection, neglect) (Tabak et al., 2011). Thus, in our study, the positive relationships among GS, EL, and oxytocin, further support a closer examination of the role oxytocin plays in gendered workplace stress and physiological dysregulation over the long-term.

Although higher levels of oxytocin have also been associated with strong affiliation and positive social bonds (Holt-Lunstad et al., 2015), researchers suggest that this paradox can be explained by viewing relational distress as signaling the need or desire for stronger social bonds (Van Anders et al., 2011). This would implicate oxytocin in the management of social relationships more generally (Van Anders et al., 2011), rather than with positive affiliations alone (e.g., tend-and-befriend). Furthermore, oxytocin appears to act as an emotional amplification (Van Anders et al., 2013) and social salience system (Shamay-Tsoory and Abu-Akel, 2015) in service of efficient social interaction, regardless of emotional valence. This suggests that our novel observation of a positive relationship between deep acting and oxytocin responses to stressful interactions is a potential mechanism linking greater emotion perception and regulation with deleterious health outcomes over the long-term. That is, more deep acting – possibly a behavioral manifestation of oxytocin release, given their positive association in our findings – is accomplished through increased sensitivity to social cues for accurate emotion perception and successful emotion reappraisal during times of stress. For example, gendered social distress may be associated with increased conciliatory deep acting that aims to diffuse volatile interactions in public-safety workplaces, such as that of the communicator. Evidence suggests these emotion-centered behavioral changes are indeed linked with oxytocin release during stressful social interactions (von Dawans et al., 2019). Thus, the positive associations between GS, EL, and oxytocin – in a field setting that necessitates repeated exposures to potentially traumatic events and presents with elevated levels of PTSD – challenges the notion that oxytocin and deep acting are inherently prosocial and protective against the negative effects of stress.

This recent recognition of oxytocin as highly relevant for human stress processes, especially social stressors, has compelled researchers to consider stress and emotion as more relationally based than traditional conceptions. Thus, the social features of the environments in which individuals and groups are operating, including institutionalized gender structures (Risman, 2018), may be profoundly important for their emotional experiences. GS, or distress experienced through adherence to stereotypical gender norms, has been established as a source of stress that is associated with negative social interactions in the workplace (Leskinen et al., 2015; Swan, 2016) and negative consequences for health and well-being like cardio vascular disease, low self-esteem, depression, shame proneness, and anxiety (Watkins et al., 1991; Bekker and Boselie, 2002; Richmond et al., 2015). Interestingly, decreased satisfaction with the quality of social relationships contributes to the development of PTSD. Those who report lower social relationship quality shortly after a traumatic exposure (and perhaps have higher associated oxytocin) may be more susceptible to the development of PTSD (Freedman et al., 2015).

There was also a significant positive association between masculine GS and RMSSD_HRV_, demonstrating greater experience of masculinity-associated stress with higher parasympathetic control in the first 30 s of an event. An increase in HRV is thought to reflect parasympathetically mediated recovery rather than sympathetically mediated stress. However, as discussed in the PTS section below, it may be that a short-term increase in RMSSD serves as a mechanism to support improved emotion perception and regulation in times of complex social stress and information gathering, especially for those with greater concerns regarding gender normativity. Research demonstrating increases in HRV during emotion perception and recognition (Dimitroff et al., 2017) or suppression and reappraisal (Butler et al., 2006) appears to support this contention. As such, our findings are in concert with others’, suggesting an initial physiological response that facilitates emotion perception and regulation through a more still, perceptive physiological state that is realized through maintained PNS engagement (Kemp et al., 2017) and release of oxytocin (Uvnäs-Moberg et al., 2005). These findings also support the social salience hypothesis for oxytocin (Shamay-Tsoory and Abu-Akel, 2015) and suggest HRV and oxytocin are key when trying to understand new social information, regardless of the emotional valence of the situation. Taken together, these data suggest that a focus on occupations that may be subject to different gendered dynamics – where gender is particularly salient – is helpful for addressing how it is that gender is a fundamental organizing feature of work that affects health. While communicators are mostly women, they work within the bounds of larger policing organizations. Policing is a hyper-masculinized organization that can pathologize emotions and push emotional work to the margins as women’s work (Shelley et al., 2011; Papazoglou, 2013; Swan, 2016).

### Posttraumatic Stress

Consistent with previous findings that communicators have considerably higher rates of PTSD symptomology than police officers and the general public (Regehr et al., 2013), 35% of this sample of communicators reported PTS symptomology consistent with a diagnosis of PTSD. PTS was positively related to cortisol AUC_G_ and event-related RMSSD_HRV_, suggesting activation of both the SNS and the PNS during these events. Because acute stress is generally associated with a decrease in HRV (activation of the SNS), this association may seem counter to typical stress responses. However, these findings suggest that during acute stress it is possible to have a short-term increase in HRV. Physiological responses such as these, during stressful events in the communicators’ workplace, might represent the increased salience of social cues, and occur in service of optimized attention to social variables, accurate emotion recognition and effective emotion regulation. Supporting this line of thinking is evidence demonstrating that, even though higher resting HRV is most often associated with better emotion regulation abilities and flexible adaptation for healthier social functioning (Balzarotti et al., 2017), some research on emotion regulation (e.g., suppression and reappraisal) suggests this stressful and effortful work is accompanied by increases in PNS activity during distress (Butler et al., 2006; Denson et al., 2011). Thus, as was also suggested for the repeated release of oxytocin, above, over the long-term, repeated exposure to social threats – although they are associated with short-term increases in HRV – may have negative consequences for health.

Cumulative exposure to certain classes of traumatic events has recently been identified as a nuanced predictor of posttraumatic stress injuries (O’Donnell et al., 2017). Given communicators’ high rate of posttraumatic symptoms, perhaps repeated workplace exposures with concurrent intense emotion work requirements, function as a particular class of chronic or traumatic exposures that make communicators uniquely vulnerable to stress-related dysregulation and disease. There is a growing body of evidence that social stress and context play a major role in the development of PTSD (Vogt et al., 2017), making oxytocin a possible mechanism linking persistent social stressors with symptom development. Exposure to chronic trauma contributes to the development of symptoms of impaired emotion regulation and relationship distress (Marinova and Maercker, 2015). Furthermore, Li et al. (2018) recently found that the highest levels of oxytocin were present in women with certain forms of PTSD and that both oxytocin and cortisol seemed reciprocally dysregulated in these women. If communicators are regularly subject to events that are associated with the release of oxytocin, and oxytocin is associated with both an increase in the salience of social cues and subsequently suppressed cortisol reactivity, this set of circumstances may make communicators more vulnerable to the negative emotional tone of events and increase their risk for developing posttraumatic stress injuries, subsequent to cumulative traumatic exposures. These findings highlight the possibility of both stress-attenuating and stress-facilitating effects of oxytocin that are likely dependent on contextual features such as EL requirements and gendered organizational stressors that are at play in the communicators’ workplace.

### Momentary Physiological Output (AUC_G_) vs. Reactivity to Acute Stressors (AUC_I_)

Area under the curve with respect to increase values for oxytocin and cortisol, unlike AUC_*G*,_ were unrelated to any of the other study variables. Although we predicted that they would both be related self-report chronic stressors, it may be that the wide range of reactivity observed in general highlights both interindividual variability and the difficulty of controlling for potentially confounding factors in our field setting. Nevertheless, researchers have not directly examined the biological basis for differentiating between overall output (AUC_G_) and change over time (AUC_I_) subsequent to stressful events (Khoury et al., 2015). Patterns in anticipatory stress may contribute to the experience of chronic stress in important ways (Engert et al., 2013; Brosschot et al., 2018). For instance, persistent maladaptive psychological processes have been associated with AUC_G_ cortisol as opposed to AUC_I_ during social stress, perhaps suggesting the utility of the AUC_G_ measure under chronically stressful conditions (Olivera-Figueroa et al., 2015). Anecdotally, communicators often spoke of tremendous amounts of anticipatory stress as an unavoidable function of being prepared for the inevitable. In order to understand these two measurements more fully, future studies would benefit from incorporating true baseline assessments as well as within-subject designs.

Additionally, our finding that AUC_G_ values for salivary cortisol and oxytocin were negatively related post-event, but differentially related to subjective experiences of EL, GS, and PTS, has important implications for research regarding the discrepancy between subjective ratings and physiological measures of stress. A review of research reporting both a measure of subjective emotional stress and physiological stress response (salivary cortisol) found that only one quarter of the studies reported a significant correlation between the two (Campbell and Ehlert, 2012). A reasonable speculation would be that oxytocin, a social salience hormone and suspected cortisol buffer, plays a key role as mediator in the lack of a consistent correlation between different subjective emotional stressors and physiological response of stress systems, including the HPA axis (Engel et al., 2019). Findings from the present study have pointed to potential mechanisms behind this weak relationship. The stimulation of oxytocin during sustained engagement throughout complex and stressful social interactions may both attenuate cortisol responses and reconfigure subjective experiences. Lending support for our contention that oxytocin release may be related to subjective experiences of complex social stress is that intranasally administered oxytocin decreases arousal ratings for threatening human stimuli (Norman et al., 2011) and increases calmness during social stress (Heinrichs et al., 2003). Future research might consider the relationship between subjective ratings of stress and simultaneous changes in oxytocin, cortisol and HRV.

### Acute Stress Response as the Embodiment of Social Relations

The associations we found in the present study suggest potential pathways for the physiological embedding of gendered, emotional social experiences. As such, we might then ask, to what extent are typical biobehavioral responses to stressors – such as tend-and-befriend or fight-or-flight – an accumulation of life experiences, such as the gendered organization of work? While oxytocin-related functioning may be central to efficient social stress processing and behavioral response, contrary to common conceptualizations, repeated release of oxytocin, alongside prolonged PNS engagement during acutely stressful events in the workplace, may have deleterious consequences for the health of communicators. Their patterned and pervasive stress pathway activation may lead to maladaptive physiological changes and dysregulation (McEwen, 2012), over the span of one’s career (Juster et al., 2013). In what little research has been conducted with communicators, their work is consistently associated with negative health outcomes such as elevated rates of burnout, depression, PTSD, obesity, and physical health complaints (Lilly and Pierce, 2013; Regehr et al., 2013; Trachik et al., 2015; Lilly et al., 2016; Steinkopf et al., 2018).

### Strengths and Limitations

The purpose of our study was to explore the associations between EL, GS, and PTS (as chronic forms of gendered work-related stress) and physiological stress responses to acutely stressful events in a police communications center. In doing so, we considered potential neurohormonal pathways linking acute and chronic social stress to deleterious health outcomes. While informative, the constraints of data-collection in a naturalistic setting led to a relatively small sample size. This limited our ability to control for or test other variables that mediate cortisol, oxytocin, and HRV, such as sex, age, birth control use, and time of day. Additionally, years of work experience as a communicator, along with the type and length of events experienced by participants, likely influenced the large range of physiological responses. However, despite the small sample size, and large range of physiological dependent variables, we were able to demonstrate that EL, GS, and PTS were each unique and significant predictors of cortisol, oxytocin and HRV in distinct ways. Future studies would benefit from larger samples allowing for detailed consideration and statistical control of other relevant factors such as age, time of day, event type, event length, and hormonal birth control and/or hormone replacement therapy.

Although the gendered nature of this profession is accurately reflected in the relative numbers of women and men, we were only able to capture stressful event responses from three men. This precluded a sex-based analysis of the data. While most studies regarding the association of oxytocin with cortisol have not found a moderating effect of sex (Brown et al., 2016; Engert et al., 2016), larger samples may allow for disaggregation for sex/gender based analyses.

The cross-sectional design prohibits the discovery of any causal mechanisms. However, the significant associations between these subjective social stressors and physiological stress response during an event are novel and relevant for understanding how social conditions are embodied through neurohormonal pathways. Future research may benefit from baseline samples in a naturalistic setting and a within subject design using multiple events, allowing for better control of interindividual differences.

## Conclusion

Gendered work, such as police communications, may be carried out in masculinized work environments, creating unique tensions and health consequences for workers. For communicators, chronic forms of gendered workplace stress, namely EL, PTS, and GS, each had differential associations with physiological responses to acutely stressful events in the workplace. The present study is the first to examine these associations in a naturalistic setting.

Our findings are important as stressful work environments can become associated with sustained threat perceptions, leading to the default stress response of generalized unsafety (Brosschot et al., 2016, 2018), resulting in stress-related disorders such as PTSD. Our study makes an important contribution to the occupational stress and trauma literatures by drawing attention to the multi-faceted nature of occupational factors and physiological responses that may put communicators at increased risk for stress injuries: EL, GS, and PTS are related to changes in cortisol, oxytocin and HRV. Furthermore, our study encourages better understanding of the links between physiological and behavioral adaptations to social conditions such as those that occur at work, exploring how regulated interactions might reproduce social divisions and gendered emotional norms (Taylor, 2012; Davis and Risman, 2015) that transform gender and work into social determinants of health. Taken together, patterns in acute changes in cortisol, oxytocin, and HRV may be used to make visible, the consequences of cumulative exposures to social stressors experienced in localized, highly gendered social contexts, such as the workplace.

## Data Availability Statement

The datasets generated for this study are available on request to the corresponding author.

## Ethics Statement

The studies involving human participants were reviewed and approved by the University of Toronto’s Health Sciences Research Ethics Board. The participants provided their written informed consent to participate in this study.

## Author Contributions

All authors contributed to the conception and design of the study, manuscript revision, read and approved the submitted version. AB collected the data, organized the database, performed the statistical analysis, and wrote the first draft of the manuscript.

## Conflict of Interest

The authors declare that the research was conducted in the absence of any commercial or financial relationships that could be construed as a potential conflict of interest.

## Footnotes

1 With thanks to sociologist Arlie Hochschild for her foundational work on emotional labor in *The Managed Heart: Commercialization of Human Feeling.*
